# The Relationship between Post-Traumatic Stress Disorder Due to Brain Injury and Glutamate Intake: A Systematic Review

**DOI:** 10.3390/nu16060901

**Published:** 2024-03-21

**Authors:** Benjamin F. Gruenbaum, Alexander Zlotnik, Anna Oleshko, Frederic Matalon, Honore N. Shiyntum, Amit Frenkel, Matthew Boyko

**Affiliations:** 1 Anesthesiology and Perioperative Medicine, Mayo Clinic, Jacksonville, FL 32224, USA; gruenbaum.benjamin@mayo.edu; 2 Anesthesiology and Critical Care, Soroka Medical Center, Ben-Gurion University of the Negev, Beer Sheva 84101, Israel; alekszl@clalit.org.il (A.Z.); fmatalon1125@gmail.com (F.M.); hnkafor@yahoo.com (H.N.S.); 3 Biology and Methods of Teaching Biology, A. S. Makarenko Sumy State Pedagogical University, 40002 Sumy, Ukraine; annaoleshko8567@gmail.com; 4 Emergency Medicine Recanati School for Community Health Professions, Ben-Gurion University of the Negev, Beer Sheva 84101, Israel; frenkela@bgu.ac.il

**Keywords:** blood–brain barrier, diet, glutamate, post-traumatic stress disorder, traumatic brain injury

## Abstract

There is a growing body of evidence that suggests a connection between traumatic brain injury (TBI) and subsequent post-traumatic stress disorder (PTSD). While the exact mechanism is unknown, we hypothesize that chronic glutamate neurotoxicity may play a role. The consumption of dietary glutamate is a modifiable factor influencing glutamate levels in the blood and, therefore, in the brain. In this systematic review, we explored the relationship between dietary glutamate and the development of post-TBI PTSD. Of the 1748 articles identified, 44 met the inclusion criteria for analysis in this review. We observed that individuals from countries with diets traditionally high in glutamate had greater odds of developing PTSD after TBI (odds ratio = 15.2, 95% confidence interval 11.69 to 19.76, *p* < 0.01). These findings may support the hypothesis that chronically elevated blood glutamate concentrations caused by high dietary intake invoke neurodegeneration processes that could ultimately result in PTSD. Further studies will clarify whether lowering glutamate via diet would be an effective strategy in preventing or treating post-TBI PTSD.

## 1. Introduction

Traumatic brain injury (TBI) is a severe public health crisis with more than 50 million cases reported worldwide annually [1], amounting to billions of dollars in associated annual costs [2]. It is widely accepted that depression, dementia, and anxiety are common neuropsychiatric complications of TBI [3,4,5,6,7]. While post-traumatic stress disorder (PTSD) was initially believed to be unrelated to brain injury [8,9,10], more recent investigations have highlighted a relationship between TBI and the development of PTSD [11,12,13,14].

A large body of evidence now confirms a tendency of PTSD occurrence after TBI [15,16,17]. In fact, some studies suggest that post-TBI PTSD is as common a psychiatric complication as post-TBI depression [18]. Several theories explaining post-TBI PTSD in individuals who are unable to recall the traumatic event have been offered [13,19,20,21,22,23,24,25].

PTSD also carries with it a high risk of comorbidities such as major depressive disorder, anxiety disorders, and substance abuse [26,27]. Unfortunately, PTSD often shows a limited response to pharmacologic treatment [28], though clinicians remain optimistic about discovering new therapies to treat its symptoms and control the risk of comorbidities. Despite these efforts, there is still a lack of a unified understanding of the mechanism of PTSD development, especially in cases associated with head trauma [29,30].

Generating and examining new mechanistic hypotheses is necessary for breakthroughs in this field and could result in the development of updated strategies for the treatment of this pathology. A recent related hypothesis points to the development of post-TBI neuropsychiatric sequelae through chronic blood–brain barrier (BBB) destruction, chronic glutamate neurotoxicity, and neurodegeneration [31,32]. In a previous investigation, we examined the effect of dietary glutamate on chronic neurotoxicity and its role in the development of post-TBI depression [33].

In this systematic review, we explore the relationship between dietary glutamate and the development of post-TBI PTSD in 321,057 patients. Specifically, we compare the incidence and prevalence of post-TBI PTSD in Asian countries with traditionally rich-glutamate diets that potentially cause an increase in blood glutamate levels relative to regions with poor-glutamate diets, including in Europe, North America, Australia, and New Zealand. We theorize that following post-TBI damage to the BBB, persistently high blood glutamate levels, worsened by dietary intake, may contribute to further neurodegeneration and potentially result in PTSD.

## 2. Materials and Methods

### 2.1. Protocol and Registration

This systematic review was conducted and reported as per the Preferred Reporting Items for Systematic Reviews and Meta-Analysis (PRISMA) statement [34]. The protocol details were registered on the PROSPERO registry for systematic review protocols (CRD42023465270) prior to execution.

### 2.2. Literature Search

A literature search was conducted between October 2023 and December 2023 using the following electronic databases: ProQuest, PubMed, Web of Science, APA PsycNET, Scopus, and the Cochrane Library. Search terms included the following: (1) Traumatic brain injury; (2) TBI; (3) PTSD; (4) Post-traumatic stress disorder; (5) Posttraumatic stress disorder; (6) Post traumatic stress disorder. Using the terms above, the search included: (#1 OR #2) AND (#3 OR #4 OR #5 OR #6). All identified articles were summarized in Excel spreadsheets. Duplicates were located and removed.

### 2.3. Inclusion Criteria

Studies were included if they met the following criteria: (1) English language, with full text available; (2) adult participants (16+) from the general population; (3) confirmed TBI; (4) a diagnosis of post-TBI PTSD; (5) TBI sustained 2 months prior to the study to ensure recovery from the acute phase; (6) representation of both sexes; and (7) published between 1 January 1900 and 30 October 2023. Only randomized controlled trials and observational studies were included.

### 2.4. Exclusion Criteria

Studies were excluded if they had the following: (1) non-physical trauma-caused injuries, including focal stroke, meningitis, drug-induced injury, tumor-engendered changes, or hypoxic injuries/oxygen loss-triggered brain damage; (2) sample size with <10 TBI patients; (3) qualitative in nature; and (4) populations involving military veterans with combat-related injuries.

Populations with combat-related TBIs were excluded in order to maximize the generalizability of the results for the civilian population, since most combat-related TBIs are caused specifically by blasts. Additionally, significant differences in contextual factors between combat injuries and civilian injuries as well as the increased potential for compensation seeking in the service sector [16,35,36] potentially affect their generalizability. Moreover, publications with combat-related TBI cases use a single database, which houses data that can be reused in multiple studies. Also, repetitive TBI and psychological stress that regularly accompany combatants can also affect outcomes. Nonetheless, we also reviewed military studies for the potential that they included disparate studies with only civilian samples.

Non-English publications, conference and poster abstracts, reviews, case reports, publications with combat-recruited study populations, comments, and letters to the editor were excluded.

### 2.5. Study Screening and Selection

The titles and abstracts of studies collected through the search process were screened independently by two contributors (AO and MB) to identify studies that were appropriate for inclusion following the stated criteria. The two contributors independently assessed the full texts of these potentially eligible studies; any disagreement between the two contributors was resolved through dialogue with a third contributor (AZ). The methodology of each paper was qualitatively assessed using the Newcastle–Ottawa Scale [37].

### 2.6. Statistical Analysis

The primary outcome was the number of cases of PTSD among TBI patients. The odds ratio was calculated as previously described [38,39,40].

## 3. Results

### 3.1. Search Results

**Figure 1:**
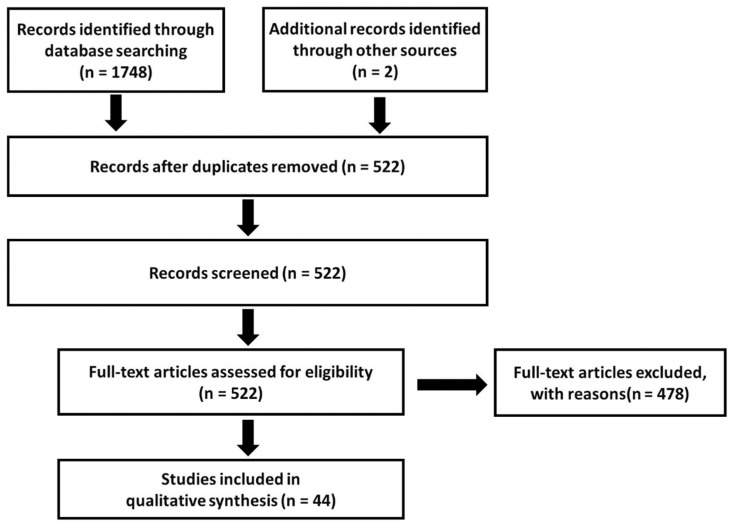
A flow chart of the study selection process.

The database searches yielded 352 hits in PubMed, 264 in ProQuest, 629 in Web of Science, 288 in APA PsycNET, 150 in Scopus, and 65 in Cochrane Library (Figure 1).

Two additional publications were identified through a manual search of the reference lists from the included publications. After the removal of duplicates, the search was left with 522 unique records. The exclusion of publications describing duplicate cohorts and groups of combatants or children reduced the total number of publications to 48. Three different publications by Bryant, R.A., et al. [19,41,42] and two publications by Bryant, R.A., et al. [43,44] published in 2000–2001 and 1998–1999, respectively, containing similar incidence rates of TBI and PTSD, were identified. Because it was likely that these papers used the same dataset, with the same patient population, only one [41] out of the three publications in the first group of publications and one [43] out of two in the second group of publications were included for analysis after consultation among the authors.

After we analyzed the data, a notice of retraction of an article by Xu et al. [45] was published. The reason for the retraction was as follows: “The investigation found evidence of systematic manipulation of the publication and review process”. Although the reason for the retraction was not falsification of data, it was clear that the article did not meet the criteria for publication in that journal. Thus, we removed the article from our review.

Consequently, the final number of publications was 44, and the final number of cases contained therein was 321,057. A full list of references of the identified publications is provided in Table S1.

### 3.2. Comparison of Study Groups

**Figure 2:**
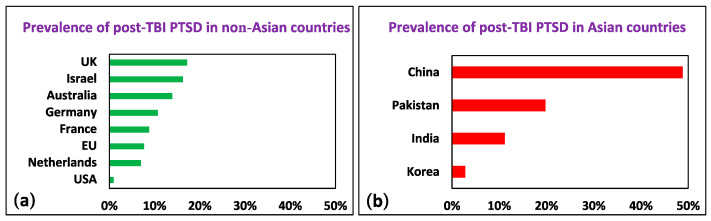
Prevalence of post-TBI PTSD in (**a**) non-Asian and (**b**) Asian countries.

We grouped the cases by region of origin. The calculated odds ratio (OR) in the Asian group was 15.2 (95% CI [11.69, 19.76]; Z = 20.33; *p* < 0.01) in comparison to the European, American, Australian, UK, and Israeli populations in the first year following TBI, supporting our hypothesis that there is a higher incidence of PTSD after TBI in Asian countries (Figure 2). As we note in the subsequent section, the differences in the assessment and diagnosis of post-TBI PTSD are significant; however, it is unlikely that these differences can completely account for the enormous discrepancy in the incidence of post-TBI PTSD among non-Asian countries relative to Asian countries (see Supplementary Materials).

### 3.3. Article Quality

**Figure 3:**
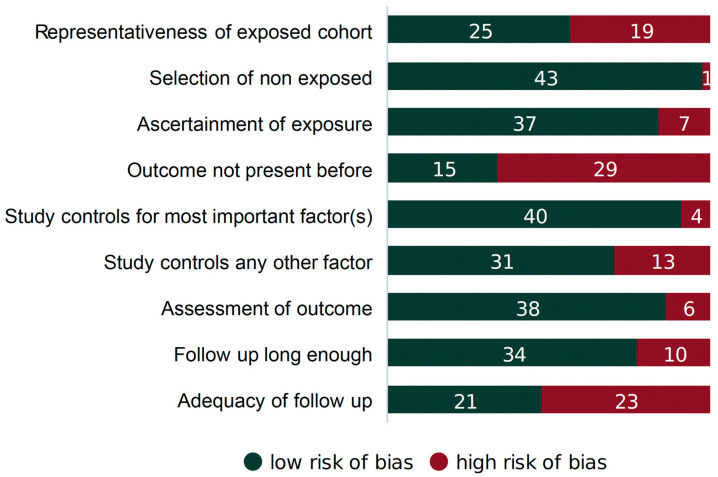
Sources of potential bias in cohort studies (*n* = 44).

Each article considered in this study underwent review for potential bias and methodological integrity using the Newcastle–Ottawa Scale [37]. This scale assesses the risk of selection bias, the comparability of comparison groups, and the validity of outcome/exposure ascertainment for observational, non-randomized investigations. The scale has specific evaluation criteria for case–control and cohort studies, and we labeled each study accordingly. Bias for every article was scored as high, low, or unclear. The results of the evaluation are summarized in Figure 3. Each article was scored independently by two reviewers (AO and FM). Any disagreement between the two reviewers was resolved via dialogue with a third reviewer (MB).

## 4. Discussion

Post-TBI PTSD involves the manifestation of typical PTSD symptoms, such as intrusive thoughts or memories, avoidance behaviors, negative alterations in mood or cognition, and heightened arousal or reactivity. In addition to TBI, risk factors of PTSD include a low education level, Black ethnicity, and injury type [4]. Other risk factors are repeated head trauma [46], being a combatant [14,46], and a history of mental illness [14]. Mild TBI demonstrably raises predicted PTSD symptoms by a factor of 1.23, while moderate or severe TBI amplifies predicted symptoms by a factor of 1.71 [18,47]. The mechanisms of post-TBI PTSD development are unclear, but it is apparent that they have multifaceted aspects. The hypothesis of the current investigation points to neurodegeneration due to the destruction of BBB and chronic glutamate neurotoxicity as one of the possible key mechanisms for the development of PTSD. Confirming this hypothesis could enable improved therapies for the treatment of this severe illness.

### 4.1. The Relationship between PTSD and TBI

The relationship between PTSD and TBI is complex and significantly impacts pathways for diagnosis and treatment. The interplay between PTSD and TBI can complicate recovery and has important implications for clinical management. Research has highlighted the co-occurrence of these conditions, particularly mild TBI, and the challenges in differentiating their symptoms. The overlap in symptoms and the bidirectional influences of PTSD and TBI on recovery have been the subject of extensive research, especially in the context of war-related injuries [46]. This research focus considerably impacts the assessment and treatment of individuals with a history of TBI, given that PTSD can exacerbate or mask many of the symptoms attributed to post-concussive symptoms. Because symptoms attributed to TBI are potentially linked to PTSD [11] or represent conditions known as “persistent symptoms after TBI” [48] and “acute stress disorder”, the coexistence of these conditions can complicate diagnosis [10]. Additionally, different approaches exist to diagnose PTSD, which can produce inconsistent diagnoses of the illness [49].

Post-TBI PTSD development is a multifaceted process. Several theories on how to elucidate the putative mechanisms underlying the development of PTSD after TBI have been suggested, including neurobiological [22,30], psychological [11], and cognitive factors [50], reflecting a potentially complex interaction between the physiological and psychological consequences of TBI [30].

Despite some understanding about the reasons for post-TBI PTSD, PTSD in general is poorly amenable to pharmacologic treatment [28]. And while clinicians retain hope of unearthing new drugs against the illness, there remains no clear consensus in the understanding of the mechanism of PTSD development that is specifically associated with head trauma [29,30]. The present inquiry provides dietary evidence that supports our hypothesis that BBB dysfunction has an important role in the development of PTSD through the mechanisms of neurodegeneration and chronic glutamate neurotoxicity.

### 4.2. The Relationship between BBB and PTSD

The association between PTSD with BBB permeability has been a subject of growing interest. BBB disruption seems to significantly influence the pathophysiology of PTSD, and PTSD is associated with increased BBB permeability, possibly contributing to the development of cognitive dysfunction and other symptoms associated with the disorder [51,52,53]. Multiple mechanisms clarifying the link between PTSD and BBB permeability have been advanced. For instance, molecular mechanisms and inflammatory signaling pathways are potentially responsible for stress-induced BBB disruption, with evidence pointing to increased BBB permeability as a crucial factor in the development of PTSD-related behavioral abnormalities [53,54]. Additionally, the role of microglia in BBB impairment and cognitive dysfunction in a PTSD-like rodent model has been highlighted, further emphasizing the potential impact of BBB integrity on the pathogenesis of PTSD [52].

Furthermore, the association between PTSD and dysfunctional endothelia has been implicated, implying that increased BBB permeability likely predisposes individuals to the development of PTSD, potentially linking the disorder to vascular and cerebrovascular conditions [51]. Moreover, the breakdown of the BBB may be involved in the early cognitive dysfunction associated with PTSD, suggesting that BBB impairment underlies the common pathological foundation of PTSD and cognitive dysfunction [52].

In summary, there is compelling evidence of a relationship between PTSD and BBB permeability as evidenced from recent studies. Increased BBB permeability conceivably has a critical impact on the incidence and manifestation of PTSD-related symptoms, including cognitive and psychiatric dysfunction. The causal connection between BBB and PTSD is still unclear. This study focused on the hypothesis that BBB dysfunction critically affects the development of PTSD. Further investigation into the mechanisms underlying BBB disruption in the context of PTSD must be conducted to advance our understanding of the disorder and explore potential therapeutic targets.

### 4.3. TBI and the Disruption of the BBB

The dysfunction of the BBB in the acute and subacute stages, which occur in the first days and weeks after TBI, is well documented [55,56]. However, the chronic stage of the TBI-related destruction of the BBB requires clarification, as it is a relatively new theory. Here, we clarify the role of long-term BBB dysfunction in the development of neurodegenerative processes after TBI.

#### 4.3.1. BBB Regulation in CNS Disorders

**Figure 4:**
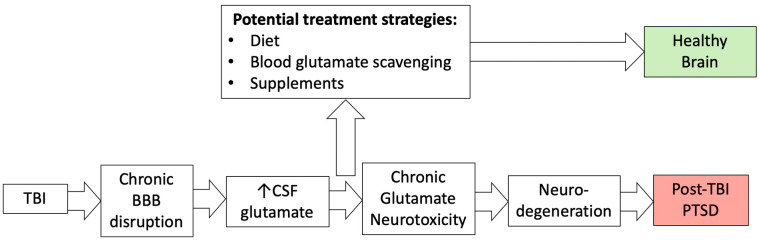
The relationship between TBI, blood–brain barrier disruption, chronic glutamate neurotoxicity, and PTSD.

Provably, BBB dysregulation correlates with the development of cerebrovascular, neurodegenerative, and neuroinflammatory diseases, including stroke, TBI, brain tumor, multiple sclerosis, Alzheimer’s disease, Parkinson’s disease, epilepsy, edema, glaucoma, amyotrophic lateral sclerosis, depression, anxiety, and dementia [57,58,59,60]. Environmental factors can also affect BBB permeability [31,61]. The proposed pathway for the development of neuropsychiatric diseases, which includes the chronic impairment of BBB permeability, glutamate neurotoxicity, and neurodegeneration, is shown in Figure 4.

Although the relationship between these illnesses and BBB disruption is apparent, its nature has not been clearly elucidated yet. In particular, it is unclear if a dysfunctional BBB stems from the pathological condition or if damage to the BBB is the main pathogenic factor that occurs before the disease onset [57]. This inquiry relies on the causal link between neurodegenerative processes in the context of the long-term impairment of the BBB permeability.

#### 4.3.2. Mechanisms of the BBB Dysfunction in TBI

BBB destruction after brain injury is referred to as biphasic [62]. The first phase reaches its zenith at 5 h after the injury [62] and the second phase at 72 h after the injury [63,64,65] in rats and occurs in humans on day 3 after the injury [66,67,68]. The recovery of a healthy BBB has been shown to take 1–3 months [69] and up to 10 months in rats [70] and up to years after injury to the brain in humans [55,71,72]. Damage to the BBB impedes the transfer of cerebral glutamate from the extracellular fluid to the bloodstream [73]. The integrity of the BBB naturally limits the pathological increase in extracellular fluid and cerebrospinal fluid glutamate levels during neuronal death and crucially maintains the upper levels of extracellular fluid and cerebrospinal fluid glutamate concentrations in the brain after TBI. In addition, BBB constantly modulates the lower threshold of brain glutamate content of the healthy as well as the impacted brain following TBI. Recently, we identified BBB permeability as a key to regulating both the high and low limits of brain glutamate in healthy and injured brains [32].

### 4.4. The Relationship between Neurodegeneration and the BBB

The relationship between neurodegeneration and BBB permeability is gaining substantial attention. Investigations have highlighted a close association between BBB dysfunction and neurodegenerative diseases, indicating a bidirectional relationship. Reportedly, a well-functioning BBB is crucial to maintaining healthy brain tissues [74]. A disruption in the BBB integrity, often linked to increased permeability, occurs in various neurodegenerative conditions, such as Alzheimer’s disease and Parkinson’s disease. This impairment can lead to the entry of harmful substances, inflammatory factors, and oxidative stress into the brain, potentially contributing to neuronal damage [75].

### 4.5. The Relationship between Neurodegeneration and PTSD

Recent research has found that the association between PTSD and neurodegenerative disorders is significant. Demonstrably, chronic PTSD is linked to a higher risk of neurodegeneration, including conditions such as dementia, Alzheimer’s disease, and Parkinson’s disease [76,77,78]. This association is supported by evidence of elevated levels of markers associated with neurodegeneration in individuals with PTSD [24]. Furthermore, clinical structural neuroimaging studies have uncovered links between PTSD and the loss of neural integrity in key brain regions, such as the hippocampus, amygdala, and prefrontal cortex, pointing to a potential connection between PTSD and neurodegeneration [79]. The duration and severity of PTSD could be linked to the extent of neurodegenerative changes [79]. Although evidence suggests a strong association between PTSD and neurodegeneration, the causal relationship between these conditions is unclear.

### 4.6. The Relationship between Neurodegeneration and Glutamate Neurotoxicity

The relationship between glutamate-induced neuronal degeneration and neurotoxicity is well established [80,81,82]. Recent discoveries about its influence on mood disorders has propelled research on updated therapies, proposing that drugs targeting the glutamatergic systems could serve as antidepressants [5,6,83,84,85,86].

Higher levels of extracellular glutamate can induce excitotoxicity by excessively activating ionotropic glutamate receptors following acute brain insults [82,87,88,89,90,91,92]. Chronic glutamate neurotoxicity has also been considered to play a role in many neurodegenerative conditions [90]. In these conditions, chronic excitotoxicity possibly appears as part of diseases where nerve cell death occurs more gradually, during which neurons encountering glutamate at higher-than-normal concentrations can eventually result in cell death [90]. Therefore, we propose that management of these neurological conditions should focus on restoring glutamatergic balance by promoting the update of glutamate and the release of extracellular glutamate.

### 4.7. Impaired BBB Permeability Disturbance in the Balance of Glutamate Concentration between the Blood and Brain Compartments

Glutamate, the brain’s most common free amino acid [93], has concentrations ranging from 50–100 μM/L in plasma and 150–300 μM/L in whole blood [31]. In the brain, concentrations can reach 10,000–12,000 μM/kg [94], while in extracellular fluids, it is much lower, between 1–10 μM/L [31]. The stable gradient between brain cells, the blood, and extracellular fluid is maintained through the facilitative and active transport mechanisms of the BBB [94]. When the BBB is healthy, it can impede the transport of glutamate between the intraparenchymal compartment and the blood compartment [73]. Several factors can contribute to an increase in post-TBI brain glutamate level, such as neuronal death [95], inflammation [96,97,98], disruptions in glutamate recycling and signaling [99], chronic stress [80], the astrocytic release of adenosine triphosphate [100], and other reasons [73,101]. We argue that the mechanisms of BBB destruction are the most important factors of increased brain glutamate as it relates to TBI [102].

### 4.8. The Role of Diet on Blood Glutamate Concentration: The Involvement of Glutamate in Neurocognitive Processes

Several factors can disrupt and cause the normally stable levels of blood glutamate concentrations to fluctuate. One of these factors may be dietary intake, caused by the presence of glutamate in many foods [33]. Traditional diets in Asian countries include high levels of glutamate because monosodium glutamate (MSG) is a popular flavoring in many cuisines, especially in East and Southeast Asian cuisine [103,104].

Many pharmacokinetic studies have pointed to the role of blood glutamate levels following dietary intake. An MSG intake of 16.0 mg/kg of body weight is a safe level; the average daily consumption in European countries and the United States sits at around 0.3–0.5 g/day [104], and up to 1.2–4 g/day in Asian countries [105]. The potential of MSG to lead to neurotoxicity, due to its role in chronic impairment of BBB permeability, is worrisome. Studies of MSG administration in food have found that blood and plasma glutamate levels rose from 1.4 to 19 times, depending on the amount of MSG consumed [106,107,108,109,110,111,112]. While MSG is considered safe for consumption by the Food and Drug Administration, several animal studies have identified potentially dangerous effects possibly associated with chronic MSG consumption [113].

Glutamate’s importance as an excitatory neurotransmitter in the central nervous system means that, when it exceeds normal levels, it can lead to excitotoxicity, associated with severe neuronal damage and disruptions to cognitive and behavioral health [104,114,115,116,117,118,119]. Recent studies have proposed relationships between the gut microbiota and the brain neurotransmitters dopamine, serotonin, GABA, and glutamate [120]. These relationships imply a correlation between dietary factors, neurotransmitter functions, and psychiatric disorders, such as PTSD. Certain studies have posited that diets with high levels of sodium glutamate raise blood glutamate and glutamic acid levels, resulting in hyper-glutamatergic neurotransmission. Hyper-glutamatergic neurotransmission has been shown with an association with several different psychiatric conditions, including PTSD [120,121,122,123,124,125]. In several studies, rodents on a diet with a high intake of sodium glutamate were observed with more depressive behaviors, such as less social interaction, anhedonia, and behavioral despair [120,126,127]. A preclinical analysis shows a possible link between sodium glutamate intake and the development of anxious and depressive-like phenotypes [120]. Animal models administered sodium glutamate showed higher rates of anxiety and behavioral dysfunction [128,129,130,131,132]. Rats with post-traumatic depressive-like symptoms and with anxiety who were treated for elevated blood and cerebrospinal fluid glutamate levels showed no difference in behavior post-treatment from that of naïve rats [5,6]. These data imply that dietary glutamate consumption and the development of PTSD have a close correlation.

In addition, glutamate dysregulation affects the pathophysiology of PTSD, which is especially relevant to glutamatergic dysfunction in trauma-related disorders. Similarly, the role of glutamate in synaptic plasticity, memory formation, and the development of PTSD-related behaviors has been hypothesized [121,122,125]. Furthermore, altered metabotropic glutamate receptor 5 (mGluR5) markers have been noted in patients with PTSD, with patient brains containing increased mGluR5 levels. This could have consequences for the treatment of PTSD, as the overstimulation of mGluR5 is linked to fear and stress-related behaviors, and drugs that moderate mGluR5 function could improve these symptoms [125]. Therefore, the relationship between consumption of dietary glutamate and the development of PTSD has been clearly outlined.

### 4.9. Current and Potential Therapeutic Strategies for Post-TBI PTSD

Treatment success rates for post-TBI PTSD vary across studies. In some studies, the success rates range extensively, with rates as high as 24% to 50% [44,133,134]. In other reports, the success rates are as low as 3% [135] or do not register the presence of PTSD at all [9,136]. Several approaches to post-TBI PTSD treatment, including cognitive processing therapy [137], prolonged exposure [137], selective serotonin reuptake inhibitors [21,138], cognitive behavioral therapy [139], eye movement desensitization and reprocessing, antidepressants, risperidone, topiramate, and even stem cells, have been met with scrutiny [30]. However, the treatment of comorbid TBI and PTSD is a complex issue, and the most effective approaches may differ depending on the individual’s specific condition and needs.

Our previous hypothesis that the integrity of the BBB is critical in controlling glutamate levels in the cerebrospinal fluid after brain injury [102] is particularly relevant after TBI, in which neurotoxicity associated with high glutamate levels induces neurodegenerative processes that ultimately lead to psychiatric disorders and, in particular, PTSD [32]. Consequently, a targeted control of elevated glutamate levels could impact the neuropsychological symptoms of acute and chronic brain diseases significantly. Therefore, chances are high that future strategies to find new therapeutic approaches will focus on the role of BBB and chronic glutamate neurotoxicity as central factors in the development of neuropsychiatric sequelae of TBI.

### 4.10. Limitations

As a systematic review, this study cannot study all the parameters of the conditions described here, since it is constrained by the methods and results of the literature it reviews. In addition, we do not explore the potential consequences of diet-related changes as a therapeutic approach for the reduction of glutamate levels. Therefore, it is our hope that future research will investigate topics that we could not include, such as comorbidities of PTSD and their relationships to glutamate, diet as a treatment for PTSD and/or glutamate dysregulation more generally, and the specific glutamate receptors that may play a role in the development of PTSD.

## 5. Conclusions

Excessive levels of blood glutamate are closely associated with the onset of PTSD following TBI. We hypothesize that the permeability of the BBB is crucial in facilitating neurodegenerative processes due to glutamate neurotoxicity, as well as the emergence of neuropsychiatric disorders. Therefore, strategies aimed at reducing blood glutamate concentrations and restoring the integrity of the BBB might aid in treating and preventing PTSD that arises after TBI. Various factors can influence blood glutamate levels, yet a functional BBB effectively shields the brain from glutamate’s neurotoxic effects. Among these factors, dietary glutamate intake appears to significantly contribute to increased blood glutamate levels. In cases of chronic conditions with BBB damage post-TBI, sustained high levels of blood glutamate, driven by high glutamate intake, can lead to brain neurotoxicity and neurodegeneration, potentially culminating in PTSD. A diet low in glutamate, along with targeted treatments for the glutamate system and dietary supplements to reduce blood glutamate, might offer effective approaches for managing PTSD following TBI.

## Data Availability

Data is contained within the article and Supplementary Materials.

## Supplementary Materials

The following supporting information can be downloaded at: https://www.mdpi.com/article/10.3390/nu16060901/s1. Table S1. Prevalence of post-TBI PTSD in Asian countries (rich-glutamate diets) vs. non-Asian countries (poor-glutamate diets). Table S2. Prevalence of post-TBI PTSD in no Asian countries. Table S3. Prevalence of post-TBI PTSD in Asian countries. References [140,141,142,143,144,145,146,147,148,149,150,151,152,153,154,155,156,157,158,159,160,161,162,163,164,165,166,167,168,169,170,171,172,173,174,175,176,177] are cited in the supplementary materials.
