# Supplementary material for: The Relationship between Post-Traumatic Stress Disorder Due to Brain Injury and Glutamate Intake: A Systematic Review
Source: Nutrients. 2024 Mar 21;16(6):901. doi: 10.3390/nu16060901 (PMC10975349; doi:10.3390/nu16060901)
Supplement: Supplementary file 1 [file nutrients-16-00901-s001.zip › nutrients-2887710-supplementary.pdf]

nutrients

MDPI

Table S1. Prevalence of post-TBI PTSD in Asian countries (rich-glutamate diets) vs. non-Asian countries (poor-glutamate diets).

Study	Population	Participant (n)		%	Reference
		TBI Total Causes	Post-TBI PTSD Causes		
Marks MR, et al. 2022	USA	207354	1232	0.59%	[140]
Albrecht JS, et al. 2017	USA	96881	269	0.28%	[141]
van der Vlegel M, 2021	EU	2864	153	5.34%	[141]
Zatzick DF, et al. 2010	USA	2228	485	21.77%	[143]
Bockhop F, et al. 2022	Germany	1776	190	10.70%	[144]
Kulbe JR, et al. 2022	USA	1143	227	19.86%	[145]
Van Praag DL, et al. 2022	EU	1134	153	13.49%	[146]
Haagsma JA, et al. 2015	Netherlands	797	42	5.27%	[147]
Stein MB, et al. 2019	USA	750	129	17.20%	[18]
Stein MB, et al. 2023	USA	714	116	16.25%	[148]
Lagarde E, et al. 2014	France	534	47	8.80%	[149]
Kosaraju S, et al. 2022	USA	504	89	17.66%	[150]
Bryant RA, et al. 2009	Australia	425	50	11.76%	[151]
Stein MB, et al. 2021	USA	421	70	16.63%	[152]
Creamer M, et al. 2005	Australia	307	32	10.42%	[153]
Haarbauer-Krupa J, et al. 2017	USA	280	75	26.79%	[135]
Scheenen ME, et al. 2017	Netherlands	262	32	12.21%	[154]
Hoffman JM, et al. 2012	USA	239	41	17.15%	[155]
Warren AM, et al. 2015	USA	231	41	17.75%	[156]
Sawyer K, et al. 2015	USA	186	25	13.44%	[157]
Alway Y, et al. 2016	Australia	149	14	9.40%	[158]
Qureshi KL, et al. 2019	UK	127	26	20.47%	[159]
Bombardier CH, et al. 2006	USA	125	14	11.20%	[160]
Gil S, et al. 2005	Israel	120	17	14.17%	[161]
Caspi Y, et al. 2005	Israel	120	22	18.33%	[162]
Hickling EJ, et al. 1998	USA	107	38	35.51%	[163]
Bryant RA, et al. 2000	Australia	96	26	27.08%	[41]
Winkler EA, et al. 2017	USA	96	28	29.17%	[164]
Alway Y, et al. 2016	Australia	85	10	11.76%	[165]
Levin HS, et al. 2001	USA	69	8	11.59%	[166]
Bryant RA, et al. 2004	Australia	68	16	23.53%	[167]
Williams WH, et al. 2002	UK	66	12	18.18%	[168]
Sumpter RE, et al. 2005	USA	66	17	25.76%	[169]
Bryant RA, et al. 1998	Australia	63	15	23.81%	[43]
Jones C, et al. 2005	UK	58	10	17.24%	[170]
Bryant RA, et al. 1999	Australia	46	9	19.57%	[171]
Glaesser J, et al. 2004	Germany	46	5	10.87%	[50]
Sumpter RE, et al. 2006	UK	34	1	2.94%	[49]
Roden-Foreman K, et al. 2017	USA	28	11	39.29%	[172]
Ohry A, et al. 1996	USA	24	8	33.33%	[173]
**Total**		**320,623**	**3805**	**1.19%**	

Table S2. Prevalence of post-TBI PTSD in no Asian countries.

Study	Population	Participant (n)		%	Reference
		TBI Total Causes	Post-TBI PTSD Causes		
Sudhakar SK, et al. 2023	India	224	25	11.16%	[174]
Zaman S, et al. 2020	Pakistan	96	19	19.79%	[175]
Choi MS, et al. 2014	Korea	71	2	2.82%	[176]
Li L, et al. 2016	China	43	21	48.84%	[177]
**Total**		**434**	**67**	**15.44%**	

Table S3. Prevalence of post-TBI PTSD in Asian countries.

	Event (PTSD)	No Event
**Population post-TBI PTSD in Asian countries**	**a**	**b**
**Population post-TBI PTSD in no Asian countries**	**c**	**d**

We calculated the confidence Interval and the odds ratio using the following formulas.

- **Odds ratio** = (a*d)/(b*c)
- **Lower 95% CI** = $e^{\ln(OR) - 1.96\sqrt{(1/a + 1/b + 1/c + 1/d)}}$
- **Upper 95% CI** = $e^{\ln(OR) + 1.96\sqrt{(1/a + 1/b + 1/c + 1/d)}}$

whereas in the publication the data were presented as a percentage of the number of cases, we recalculated and presented the data as an odds ratio.

Odds ratio was calculated as previously described [33,38].
